# Flowering Time Diversification and Dispersal in Central Eurasian Wild Wheat *Aegilops tauschii* Coss.: Genealogical and Ecological Framework

**DOI:** 10.1371/journal.pone.0003138

**Published:** 2008-09-04

**Authors:** Yoshihiro Matsuoka, Shigeo Takumi, Taihachi Kawahara

**Affiliations:** 1 Fukui Prefectural University, Matsuoka, Eiheiji, Yoshida, Fukui, Japan; 2 Laboratory of Plant Genetics, Graduate School of Agricultural Science, Kobe University, Nada-ku, Kobe, Japan; 3 Graduate School of Agriculture, Kyoto University, Muko, Kyoto, Japan; American Museum of Natural History, United States of America

## Abstract

Timing of flowering is a reproductive trait that has significant impact on fitness in plants. In contrast to recent advances in understanding the molecular basis of floral transition, few empirical studies have addressed questions concerning population processes of flowering time diversification within species. We analyzed chloroplast DNA genealogical structure of flowering time variation in central Eurasian wild wheat *Aegilops tauschii* Coss. using 200 accessions that represent the entire species range. Flowering time measured as days from germination to flowering varied from 144.0 to 190.0 days (average 161.3 days) among accessions in a common garden/greenhouse experiment. Subsequent genealogical and statistical analyses showed that (1) there exist significant longitudinal and latitudinal clines in flowering time at the species level, (2) the early-flowering phenotype evolved in two intraspecific lineages, (3) in Asia, winter temperature was an environmental factor that affected the longitudinal clinal pattern of flowering time variation, and (4) in Transcaucasus-Middle East, some latitudinal factors affected the geographic pattern of flowering time variation. On the basis of palaeoclimatic, biogeographic, and genetic evidence, the northern part of current species' range [which was within the temperate desert vegetation (TDV) zone at the Last Glacial Maximum] is hypothesized to have harbored species refugia. Postglacial southward dispersal from the TDV zone seems to have been driven by lineages that evolved short-flowering-time phenotypes through different genetic mechanisms in Transcaucasus-Middle East and Asia.

## Introduction

The timing of flowering is a reproductive trait that has significant impact on fitness in plants. Physiological and molecular mechanisms that control floral change in response to environmental conditions have been the target of recent intensive studies. In model plant systems, a group of genes that control the transition from vegetative to reproductive phases has been identified [1]–[2]. Flowering phenology is coordinated with the seasonal cycle by a complex floral gene network that uses multiple pathways and deals quantitatively with environmental signals such as photoperiod and temperature.

Natural populations of widespread plant species often show extensive variation in flowering time [3]–[5]. In contrast to recent advances in understanding the molecular basis of floral transition, very little is known about flowering time diversification within species. For example, analyses of natural flowering time variation have detected geographic clines that may result from ecological adaptation to local environments [3], [5]. Such cases give rise to a series of questions regarding intraspecific processes of flowering time diversification: How is flowering time variation structured? What are the phylogenetic relationships between early-, intermediate- and late-flowering populations? How does the geographic pattern of flowering time variation reflect the process of species dispersal? To date, few empirical studies have addressed these questions.

Because flowering time is an important reproductive trait, genealogical and ecological approaches to studying flowering time diversity may provide insights into the history of species adaptation. For this reason, we analyzed the genealogical structure of natural flowering time variation in wild wheat *Aegilops tauschii* Coss. This diploid selfing species is widely distributed in central Eurasia, the center of its distribution being in the southern coastal region of the Caspian Sea and Azerbaijan [6]. From the center, the species' natural habitats spread eastward to western China via the Kopet Dag Mountains of Turkmenistan and westward to central Syria via the valleys of mountainous southeastern Turkey. The latitudinal distribution ranges roughly from 30°N to 45°N. Within its distribution range, *Ae. tauschii* is adapted to diverse environments including sandy seashore, margins of deserts, stony hills, steppe, wastelands, roadsides and humid temperate forests [6]. It also grows as a weed in wheat and barley fields. Agronomically, *Ae. tauschii* is known as the D genome progenitor of hexaploid common wheat *Triticum aestivum* L. [7]–[8].

Daylength and winter coldness are key environmental signals that affect flowering time in many temperate plant species. Wheat is a long-day plant and extended exposure to low temperature promotes flowering in autumn-sown varieties that require vernalization. Genetically, pathways that include the vernalization (*Vrn*) and photoperiod (*Ppd*) genes control the transition from vegetative to reproductive phase [9]. Recent studies showed that natural allelic variation in the *Vrn* genes is associated with the elimination of vernalization requirement [10]–[11]. Similarly, natural allelic variation in the *Ppd-D1* gene is associated with photoperiod insensitivity [12]. The evolution of spring-sown early-flowering wheat varieties, therefore, involved genetic change in the *Vrn* and *Ppd* genes. In *Ae. tauschii* and other wild wheat species, however, the role of those vernalization and photoperiod genes in flowering time diversification is yet to be addressed.

In this study, we examined the genealogical structure of flowering time variation in *Ae. tauschii*. For this purpose, intraspecific chloroplast DNA variation analyses and a common garden/greenhouse experiment were conducted using a species-wide sample of 200 accessions. Extensive flowering time variation was observed and significant species level latitudinal and longitudinal clines were detected. Late- and early-flowering accessions were from northern and southern (especially southeastern) habitats, respectively, whereas intermediate-flowering accessions were widespread through the species' range. Chloroplast DNA genealogical analyses indicated that short-flowering-time phenotypes evolved in two intraspecific lineages. In addition, on the basis of statistical analyses that incorporated climatological data, different genetic mechanisms are proposed for the evolution of short-flowering (i.e., early-flowering and early-intermediate-flowering) phenotypes in the western (i.e., Transcaucasus-Middle East) and eastern (i.e., Asia) parts of the species range. In *Ae. tauschii*, the genealogical and ecological framework of natural flowering time variation is complex. We discuss the implications of this complexity for the process of species dispersal.

## Materials and Methods

### Plant materials

**Figure 1:**
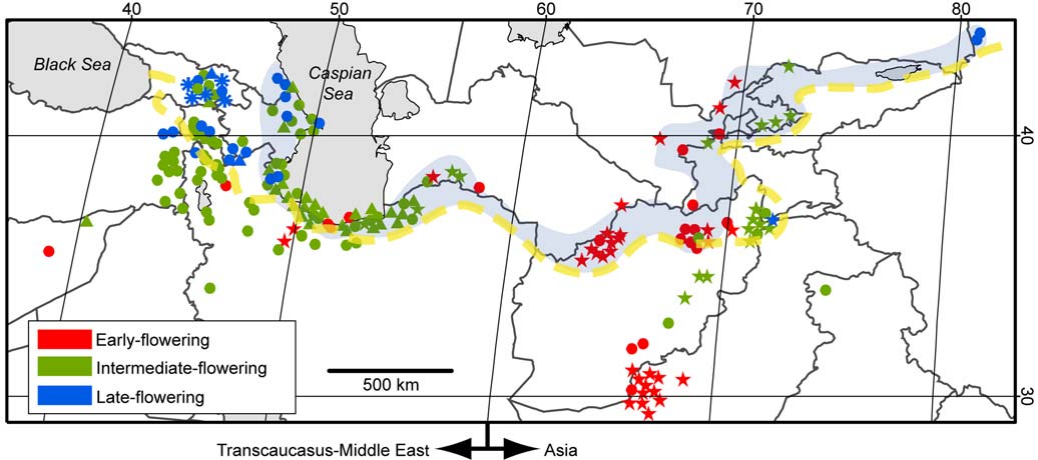
Geographic distribution of the 200 *Ae. tauschii* accessions. Circles, triangles, stars, and asterisks denote, respectively, accessions of the HG7 lineage, HG9 lineage, HG16 lineage, and HG17 lineage. For each accession, flowering time phenotype is colored according to the key. The belt area where fairly undisturbed habitats exist is shaded [25]–[26]. The dashed yellow line indicates the southern limit of the temperate desert vegetation zone at the Last Glacial Maximum [28]. The species distribution was divided into two regions in the analyses: Transcaucasus-Middle East (longitude <60°E) and Asia (longitude ≥60°E).

Two hundred accessions representing the entire natural habitat range of *Ae. tauschii* Coss. (syn. *Ae. squarrossa* L.) were used (Fig. 1). The seeds of those accessions were provided by gene banks (Table S1). For each accession, we used seeds propagated by selfing from a single plant. When geographical coordinates of the sampling sites were not available in the passport data, we estimated latitude and longitude by means of Kashmir 3D software (available at http://www.kashmir3d.com/) on scanned paper maps (scales 1∶4,000,000–1∶1,000,000) based on the locality information provided by the gene banks.

### Chloroplast DNA variation analysis

In total, 34 chloroplast DNA (cpDNA) sites were used to analyze the genealogy of *Ae. tauschii*. Of those, 21 biallelic sites were used to define haplogroups (Table S2). These sites were located in either microsatellite-flanking regions (WCt6, WCt10, WCt17, and WCt24) [13] or intergenic spacers [one between the *trn*L (UAA) and *trn*F (GAA) genes and the other between the *trn*T (UGU) and *trn*L (UAA) genes]. We used our previous studies [14]–[15] to provide data for the microsatellite-flanking-region sites in 74 accessions. New analyses provided variation data for 126 accessions. For the intergenic-spacer sites (*trn*LFb, *trn*TLa, and *trn*TLb), new data were obtained for all 200 accessions. Variations at two non-microsatellite sites (WCt24d and *trn*LFc) were not used to define haplogroups (see Results). Repeat-number-variation data at 11 cpDNA microsatellite loci (WCt1, WCt4, WCt5, WCt6a, WCt6b, WCt8, WCt10a, WCt12, WCt17a, WCt19, and WCt24a) were obtained for 138 accessions belonging to the haplogroups 7 and 16 (Table S1 and S3). Total DNA extraction and data collection were done following [14]. For the intergenic spacers, primers described by [16] were used for PCRs and sequencing.

### Common garden/greenhouse experiment

The experiment was repeated thrice (twice in Fukui [36°06′N, 136°06′E] and once in Kobe [34°43′N, 135°13′E]) using a set of 200 plants (one plant per accession) under vernalization conditions. In Fukui, seeds were sown in December of 2004 and 2005, and plants were grown individually in pots in a greenhouse. Each year, pots were arranged randomly but not rotated regularly. To reduce possible border effects, the mean of replicated days-to-flowering data was used for each accession (see below). The greenhouse was heated a little above ambient for a few weeks to enhance germination but was unheated thereafter. The monthly means of minimum daily greenhouse temperatures were 2.6°C (January, 2005), 0.6°C (January, 2006), 2.6°C (February, 2005), 1.5°C (February, 2006), 4.1°C (March, 2005), and 3.2°C (March, 2006). In Kobe, seeds were sown in November of 2004, and plants were grown under field conditions. The monthly means of minimum daily field temperatures were 2.9°C (December, 2004), 3.0°C (January, 2005), 3.0°C (February, 2005), and 5.5°C (March, 2005). At both sites, seeds (three to four per accession) were sown directly in the soil. After germination, extra individuals were removed and plant growth monitored on a regular basis. For each plant, the day of germination (i.e., the day of coleoptile emergence) and the day of flowering (i.e., the day of the first floret opening) were recorded. Flowering time was defined as the number of days from germination to flowering.

### Statistical analysis

Clustal W ver. 1.8 [17] was used for nucleotide sequence alignment, Network software ver. 4.112 (available at www.fluxus-engineering.com) [18] for reduced median network construction, Microsat ver. 1.5c [19] for calculating the genetic distance, Phylip ver. 3.57 [20] for neighbor-joining tree construction, and DIVA-GIS ver. 5.4 [21] for estimating local climatological statistics (annual precipitation and mean temperature of coldest quarter) based on the WORLDCLIM current climate data set (ver. 1.4, resolution 2.5 minutes). Principle component analysis (PCA) and other statistical calculations were done with JMP software ver. 5.1.2 (SAS Institute).

PCA was performed with the among-haplotype-correlation matrix that was obtained from the variations at 14 loci (Table S3). To calculate the matrix, the AAAAATAAAAA allele at the WCt24a microsatellite locus was treated as a stretch of 11 adenine residues. The alleles of each biallelic non-microsatellite locus (Wct6d, Wct24d, and *trn*LFc) were indicated by numerical values: “1” for the first allele and “2” for the second. In the neighbor-joining tree construction, the same 14 loci were used to calculate the log-transformed proportion of shared allele distances between haplotypes. A majority-rule consensus tree was obtained from 1,000 bootstrapped trees.

## Results

### Chloroplast DNA haplogroups and intraspecific lineages

The genealogy of *Ae. tauschii* was examined by cpDNA variation analysis. Because *Ae. tauschii* is selfing, its uniparentally-inherited chloroplast genome provides useful markers for tracing intraspecific genealogical divergence [14]. We used 21 biallelic base-change and minisatellite sites to analyze the cpDNA haplogroups in the 200 accessions (Table S2). In our preliminary analyses, the biallelic site *trn*LFc (Table S3) was polymorphic in five of 18 haplogroups defined by the 21 sites mentioned above, indicative that this locus underwent recurrent pendulum-like allelic changes from TTTTAAA to TTTAAAA and from TTTAAAA to TTTTAAA (data not shown). The WCt24d site (Table S3) had multiple alleles in the 200 accessions (data not shown). We therefore defined the haplogroups using those 21 biallelic sites at which mutation was a putatively unique event.

**Figure 2:**
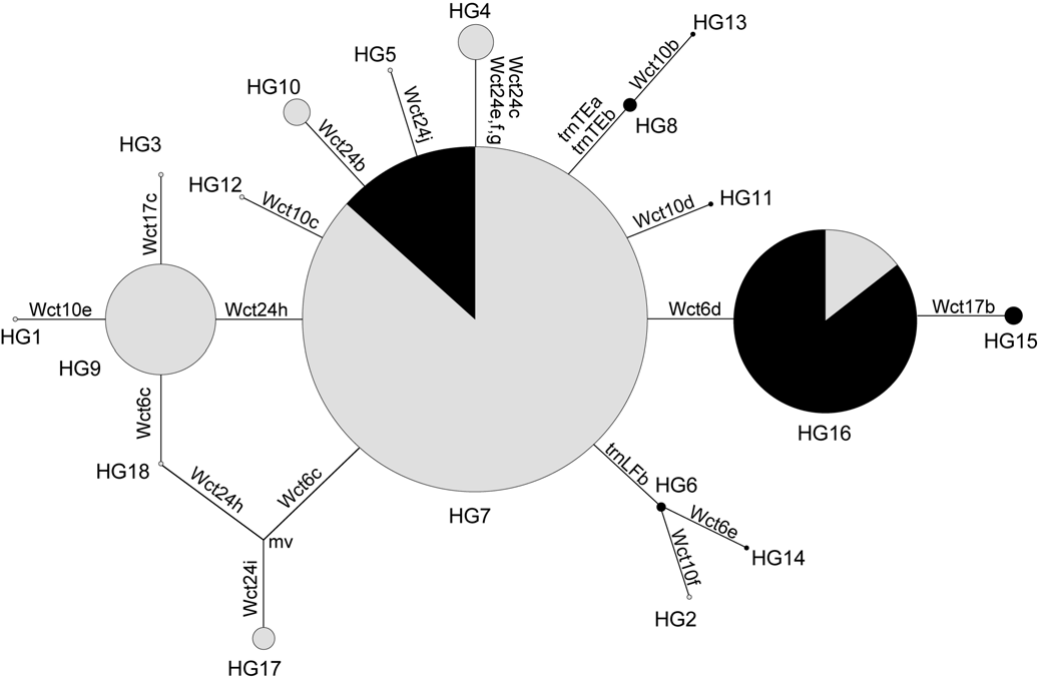
Reduced-median network for chloroplast DNA haplogroups. In total, 21 biallelic sites were used to define the haplogroups (Table S2). Circles denote haplogroups (with diameters proportional to the number of accessions). Mutations that define haplogroups are shown on the branches. mv denotes a hypothetical haplogroup not found in this study. Grey scale indicates geographic origin proportions: grey for western habitats (longitude <60°E) and black for eastern habitats (longitude ≥60°E).

Eighteen haplogroups (HGs) were identified, and a star-shaped network was obtained by reduced-median network construction (Fig. 2). Except for HG7 and HG16, haplogroups were confined either to western (longitude<60°E) or eastern (longitude ≥60°E) habitats. Consistent with our previous findings [14], chloroplast haplogroups were more diverse in western habitats (12 haplogroups) than in eastern ones (eight haplogroups). Of the three major haplogroups (HG7, HG9, and HG16), HG7 was located at the center of the network. In addition, HG7 had a wide geographic distribution, whereas HG9 was specific to western habitats (mainly the Caspian coastal region) and HG16 was distributed mainly in eastern habitats. These facts suggest that HG7 was ancestral to HG9, HG16 and many of the other minor haplogroups. Of the three major haplogroups, HG16 was unique in that its distribution center is in the eastern habitats (Fig. 2). On the basis of the network topology, we defined four intraspecific lineages: the HG7 lineage (HG7 and its derivatives HG2, HG4, HG5, HG6, HG8, HG10, HG11, HG12, HG13, and HG14), HG9 lineage (HG9 and its derivatives HG1, HG3, and HG18), HG16 lineage (HG16 and its derivative HG15), and HG17 lineage (HG17).

### Flowering time variation: geographic clines and genealogical structure

Complete three environment point data of flowering time were obtained for 198 accessions. For two accessions (KU-2083 and KU-2107), one data point was missing due to unhealthy growth. Germination was fairly uniform and occurred in most accessions about one week after sowing in all environments. The average number of days to flowering was 157.3 for the Fukui 2004 environment, 157.1 for the Fukui 2005 environment, and 169.5 for the Kobe 2004 environment. Within accessions, days to flowering varied moderately depending on the environment, the average maximum-minimum difference being 13.6 days (range, 1.0–21.0 days). Despite the fluctuation within accessions, high correlations were observed between environments (*r* = 0.90–0.91, *P*<0.0001), indicating that the observed pattern of flowering time variation has genetic bases and that the accessions responded to environmental differences in a systematic manner. Accordingly, we used the mean of the replicated days-to-flowering data as the flowering time for each accession in the subsequent analyses.

**Figure 3:**
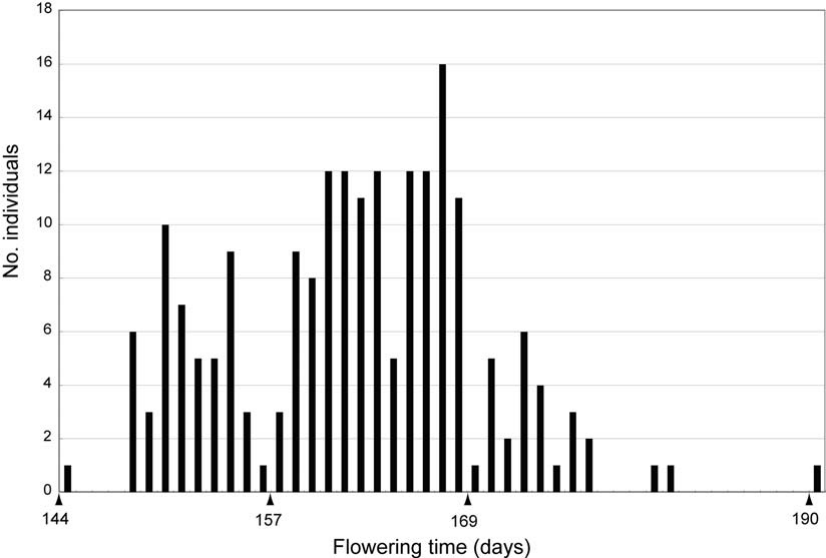
Flowering time variation in the 200 *Ae. tauschii* accessions. Flowering time for each accession is the mean of thrice-replicated data of days from germination to flowering.

**Figure 4:**
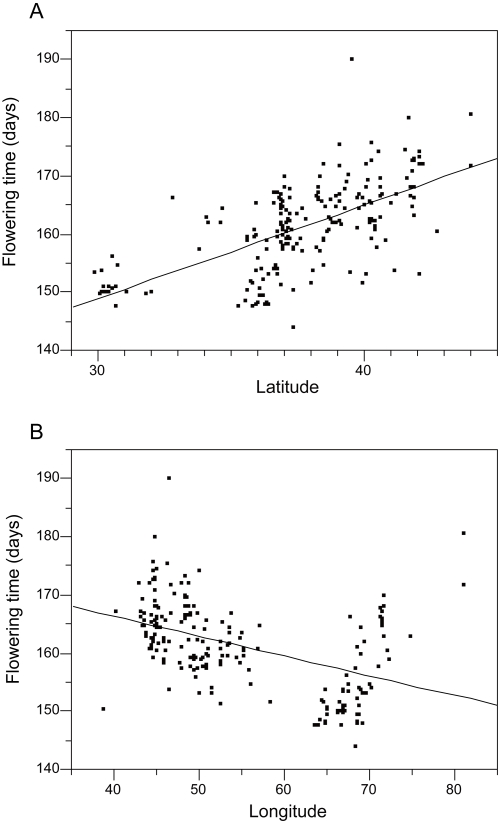
Geographic clines of flowering time. A) The relationship between latitude of origin and flowering time (regression coefficient±SE = 1.61±0.14, *P*<0.0001). B) The relationship between longitude of origin and flowering time (regression coefficient±SE = −0.34±0.05, *P*<0.0001).

Flowering time (i.e., the mean of the three data points) among accessions varied from 144.0 to 190.0 days (mean = 161.3 days). On the basis of flowering time variation (Fig. 3), we categorized the 200 accessions into three phenotypic groups: early-flowering (flowering time <157.0 days, 51 accessions), intermediate-flowering (157.0 days≤flowering time <169.0 days, 122 accessions), and late-flowering (flowering time ≥169.0 days, 27 accessions). Geographically, longitudinal and latitudinal clines were recognizable, with most early-flowering accessions from southern (especially southeastern) habitats and late-flowering accessions from northern habitats (Fig. 1 and 4). The intermediate-flowering accessions were widely spread across the species' range. These results were consistent with [22], in which the authors examined the growth habit of 31 accessions collected from Iran, Afghanistan, and Pakistan and found that early-flowering populations occur in Afghanistan and Pakistan. Accordingly, we assessed the significance of these clines by multiple regression analysis using latitude and longitude as independent variables and flowering times as the response variable. This approach was taken because there was a correlation between latitude and longitude for the origin of accessions (*r* = −0.45, *P*<0.0001). Significant effects on flowering time were detected for both latitude (*P*<0.0001) and longitude (*P* = 0.0003) (Table 1).

**Table 1:** Multiple regression analysis of the effects of latitude and longitude on flowering time.

Source	Parameter estimate	Standard error	*t* statistic	*P*
Latitude	1.35	0.16	8.62	<0.0001
Longitude	−0.17	0.04	−3.72	0.0003

Total sample size *n* = 200.

Genealogical structure of flowering time variation showed skewed frequency distributions of haplogroups (Table 2). Thirty-two of 51 early-flowering accessions (62.7%) were of the HG16 lineage. In contrast, 77 of 122 intermediate-flowering accessions (63.1%) and 19 of 27 late-flowering accessions (70.4%) were of the HG7 lineage. The HG9 lineage was phenotypically uniform, with 26 out of 28 accessions (92.9%) having the intermediate-flowering phenotype. All five accessions of the HG17 lineage had the late-flowering phenotype. The early-flowering phenotype, therefore, was unique in that the majority of the accessions were of the HG16 lineage. In the intermediate- and late-flowering phenotypes, the accessions of the HG7 lineage were common.

**Table 2:** Genealogical structure of flowering time variation.

Lineage and haplogroup	Phenotype			Total
	Early (*n* = 51)	Intermediate (*n* = 122)	Late (*n* = 27)	
HG7 lineage				
HG2	1			1
HG4	2	6		8
HG5		1		1
HG6			2	2
HG7	13	60	17	90
HG8	2	1		3
HG10		6		6
HG11		1		1
HG12		1		1
HG13	1			1
HG14		1		1
HG9 lineage				
HG1		1		1
HG3		1		1
HG9		23	2	25
HG18		1		1
HG16 lineage				
HG15	4			4
HG16	28	19	1	48
HG17 lineage				
HG17			5	5

Blanks indicate zero sample.

### Genealogical framework for the evolution of the early-flowering phenotype in eastern habitats

The early-flowering accessions were grouped in the HG7 and HG16 lineages (Table 2), suggesting that the populations of the two major haplogroups HG7 and HG16 played a role in the evolution of early-flowering phenotype. The progenitor-descendant relationship between HG7 and HG16 provided an opportunity to further investigate the evolution of early-flowering phenotype. In the course of intraspecific lineage diversification, HG16 might have diverged from HG7 in the west and independently migrated to the east. Alternatively, HG16 might have diverged from HG7 in the east. Evaluating these possibilities could provide insights into the intraspecific process for the evolution of the early-flowering phenotype.

**Figure 5:**
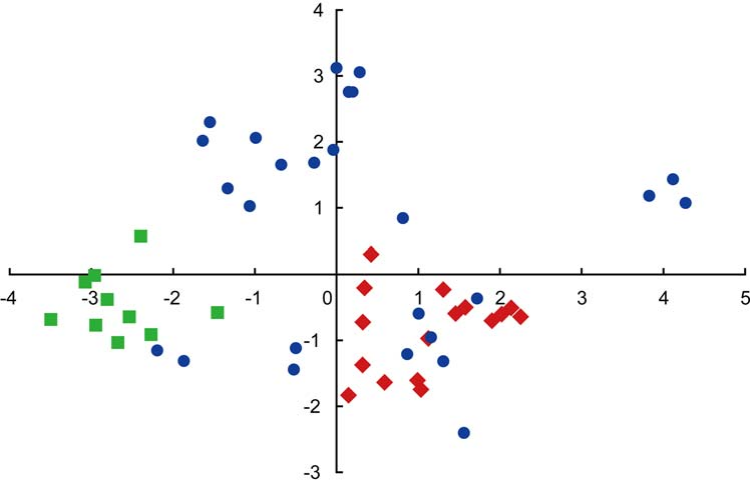
Graph of the first two axes from a principle component analysis. The fifty-two HG7 and HG7 haplotypes based on 14 polymorphic loci were used (Table S3). The first component (x) explains 25.3% and the second (y) 14.5% of the total variation. Circles, squares, and diamonds indicate the western HG7 haplotypes, eastern HG7 haplotypes, and HG16 haplotypes, respectively.

We addressed the origin of HG16 by PCA based on the variations at 11 cpDNA microsatellite loci, one multiallelic base-change locus Wct24c, the biallelic *trn*LFc locus, and the haplogroup-defining locus Wct6d. These 14 loci were so variable that they could be used to define the haplotypes in HG7 and HG16. In total, 52 haplotypes were identified in the 138 HG7 and HG16 accessions (Table S1 and S3). A PCA showed that HG16 is more closely related to the western HG7 than to the eastern HG7. In the graph of the first two axes from the PCA, the HG16 haplotypes were separated from all eastern HG7 haplotypes but fully overlapped with the western HG7 haplotypes (Fig. 5). A neighbor-joining tree based on those 14 loci had relatively weak structure but showed the same pattern (Fig. S1), indicating that the HG7-HG16 relationship detected in the PCA was resulted irrespective of treating the AAAAATAAAAA allele as a stretch of 11 adenine residues (see Materials and Methods). All this evidence supported the first scenario for the origin of HG16: HG16 diverged from HG7 in the west and independently migrated to the east. This negated the view that the HG16 early-flowering accessions are the direct descendants of the eastern HG7 early-flowering populations. From the data, however, it was difficult to infer exactly where the HG16 early-flowering phenotype originated.

### Environmental factors for flowering time diversification

Geographic clines of genetic traits may be caused by adaptation to local environments (e.g., [23]). Our statistical analysis provided evidence for significant longitudinal and latitudinal clines of flowering time in *Ae. tauschii*. To further investigate local patterns of flowering time diversification, we divided the species distribution into Transcaucasus-Middle East (longitude <60°E) and Asia (longitude ≥60°E) and tested the effects of longitude and latitude on flowering time in each region by multiple regression analysis. Contrasting patterns emerged. In Transcaucasus-Middle East, there was a significant effect of latitude (*P*<0.0001) but not of longitude (*P* = 0.14). In Asia, there was a significant effect of longitude (*P*<0.0001) but not of latitude (*P* = 0.94). These results suggested that environmental factors affecting flowering time variation are different in the western and eastern parts of the species' range.

To determine whether observed latitudinal and longitudinal flowering time clines may be caused by adaptation to local climates, a multiple regression analysis was done for each region using longitude, latitude, and two climatological statistics (estimated annual precipitation and mean temperature of coldest quarter at each sampling site) as the independent variables and flowering time as the response variable (Table 3). In Transcaucasus-Middle East, only latitude had a significant effect on flowering time (*P*<0.0001), suggesting that environmental factors other than precipitation and winter temperature affected the clinal pattern of flowering time variation. In Asia, however, longitude (*P*<0.0001) and mean temperature of coldest quarter (*P*<0.0001) had significant effects on flowering time. The multiple regression analysis showed that, in Asia, the early-flowering populations tend to occur under warm winter conditions, whereas the intermediate- and later-flowering populations tend to occur under moderate and severe winter conditions, respectively.

**Table 3:** Multiple regression analysis of the effects of latitude, longitude, mean temperature of coldest quarter (MTCQ), and annual precipitation (AP) on flowering time in Transcaucasus-Middle East and Asia.

Region (sample size)	Source	Parameter estimate	Standard error	*t* statistic	*P*
Transcaucasus-Middle East (*n* = 135)	**Latitude**	**1.83**	**0.25**	**7.31**	**<0.0001**
	Longitude	−0.18	0.16	−1.13	0.26
	MTCQ	0.00	0.13	0.04	0.97
	AP	−0.00	0.00	−0.46	0.64
Asia (*n* = 65)	Latitude	−0.18	0.14	−1.26	0.21
	**Longitude**	**1.21**	**0.17**	**6.90**	**<0.0001**
	**MTCQ**	**−0.73**	**0.12**	**−6.08**	**<0.0001**
	AP	−0.01	0.00	−1.77	0.08

Significant effects are shown in bold.

Finally, we tested by multiple regression analysis whether response to winter temperature differed between the two major haplogroups HG7 and HG16 in Asia (Table 4). For both haplogroups, a significant effect of mean temperature of coldest quarter was detected (*P* = 0.0023 for HG7 and *P* = 0.0007 for HG16). This result indicated that winter temperature was a common factor affecting the clinal pattern of flowering time variation in HG7 and HG16.

**Table 4:** Multiple regression analysis of the effects of latitude, longitude, mean temperature of coldest quarter (MTCQ), and annual precipitation (AP) on flowering time in Asian HG7 and HG16.

Haplogroup (sample size)	Source	Parameter estimate	Standard error	*t* statistic	*P*
HG7 (*n* = 12)	Latitude	−0.10	0.66	−0,15	0.89
	Longitude	−0.63	0.83	−0.76	0.47
	**MTCQ**	**−1.55**	**0.33**	**−4.66**	**0.0023**
	AP	−0.01	0.01	−1.76	0.12
HG16 (*n* = 41)	Latitude	−0.10	0.15	−0.68	0.50
	**Longitude**	**1.22**	**0.28**	**4.36**	**0.0001**
	**MTCQ**	**−0.61**	**0.17**	**−3.69**	**0.0007**
	AP	−0.00	0.00	−0.77	0.45

Significant effects are shown in bold.

## Discussion

We showed that (1) there are four cpDNA-defined intraspecific lineages (the HG7, 9, 16, and 17 lineages) in *Ae. tauschii*, (2) *Ae. tauschii* has extensive natural flowering time variation, (3) there exist significant longitudinal and latitudinal clines of flowering time at the species level, (4) the early-flowering phenotype evolved in the HG7 and HG16 lineages, (5) in Asia, winter temperature was an environmental factor that affected the longitude-clinal flowering time variation, and (6) in Transcaucasus-Middle East, some latitudinal factors affected the geographic pattern of flowering time variation. Obviously, the genealogical and ecological framework of flowering time diversification is rather complex in *Ae. tauschii*. The findings, however, shed some light on the species' history of dispersal and adaptation, as discussed below.

### Dispersal of *Ae. tauschii* in central Eurasia: an hypothesis

The putative primary region of origin of the genus *Aegilops* is in the Transcaucasus, and the diploid species of the genus radiated there 2.5–4.5 million years ago [6], [24]. From the Transcaucasus, *Ae. tauschii* dispersed eastward to western China across northern Iran and central Asia and southwestward to central Syria. Currently, *Ae. tauschii* populations growing in fairly undisturbed habitats can be seen in the belt area that extends from the Azerbaijan-Iran Caspian coast to the Alma Ata region of Kazakhstan and western China (Fig. 1) [25]–[26]. The center of species' genetic diversity exists in the Transcaucasus and Azerbaijan-Iran Caspian regions [27].

When *Ae. tauschii* established its current natural species range is not known. According to recent palaeoclimatic studies [28], the belt area was within the temperate desert vegetation zone at the Last Glacial Maximum (25,000–15,000 BP) (Fig. 1). In temperate desert vegetation, survival of *Ae. tauschii* populations may have been possible. In contrast, the southern *Ae. tauschii* habitats outside the belt area (those in Turkey, Syria, non-Caspian Iran, and the Afghanistan-Pakistan border area) were within the tropical semi-desert vegetation zone where, due to aridity, the species survival was unlikely [28]–[29]. These considerations suggest that the temperate desert vegetation zone (TDV zone hereafter) harbored glacial refugia in the late Pleistocene. This view is consistent with findings that *Ae. tauschii* is genetically, morphologically and ecologically most diverse within the TDV zone [6], [15], [27]. *Ae. tauschii* likely dispersed from the TDV zone in the postglacial era and the southern habitats were established as the environment experienced change to humid conditions in the Holocene [30]. Because *Ae. tauschii* has a weedy growth habit, human disturbance may have contributed to population migrations after the beginnings of agriculture 10,000 years ago.

That cpDNA diversity is high within the TDV supports the view that the zone harbored glacial refugia. Ten of the 18 haplogroups (HG 1,2,3,5,6,11,12,15,17, and 18) were specific to the TDV zone, whereas only two (HG13 and 14) were specific to the southern habitats (Table S1). Flowering time varied from the earliest (144 days) to the latest (190 days) in the TDV zone, indicative that this trait underwent phenotypic diversification in response to various local conditions in the course of the long-distance eastward dispersal from the Transcaucasus to Central Asia. The eastward dispersal was driven by the HG7 and HG16 lineages. HG6 and HG14 of the HG7 lineage reached the northeastern (western China) and eastern (Kashmir) limits of species' range, respectively. HG16 arose from HG7 in western habitats, migrated to the east and gave rise to HG15.

### Postglacial colonization of the southern habitats

The observed geographic and genealogical patterns of flowering time variation have implications for our view of postglacial species dispersal. The early-flowering populations are widespread in the eastern part of the species range and particularly common in the Afghanistan-Pakistan border area. This indicates that the early-flowering phenotype is well adapted to the continental Asian climate. Because there are many early-flowering HG7 and HG16 populations in the TDV zone (Fig. 1), the Asian early-flowering populations might have originated somewhere in that zone and then colonized the warm low latitudinal Afghanistan-Pakistan border area through postglacial dispersal. Alternatively, the early-flowering populations may have originated outside the TDV zone in the course of postglacial southward dispersal and then migrated back to the TDV zone through natural and/or anthropological process. The existence of HG13 (early-flowering) in the Afghanistan-Pakistan border area indicates that southeastward dispersal from the TDV zone was driven by both HG7 and HG16 lineages, whereas the HG16 populations are currently prevalent.

In common wheat, loss of vernalization requirement results in expression of early-flowering phenotypes [11]. In fact, vernalization-insensitive spring-sown (i.e., early-flowering) varieties evolved through mutations in the *Vrn* genes [10], [31]. The exact mechanism underlying expression of the early-flowering phenotype is not known for *Ae. tauschii*. The significant negative correlation between flowering time and winter temperature, however, suggests that, in Asia, the early-flowering phenotype evolved through change in the degree of vernalization requirement. Accordingly, the *Vrn* genes were probably involved in the evolution of early-flowering *Ae. tauschii*. Because HG16 is not the direct descendant of the eastern HG7, independent *Vrn* gene mutations might be responsible for the emergence of early-flowering phenotypes in the HG7 and HG16 lineages, assuming that selfing limited gene flow under natural conditions.

The Middle Eastern habitats were colonized through relatively short-distance dispersal from the Transcaucasus, but the flowering-time phenotypes are diverse in the western region: the variation range in western habitats (39.7 days) covers 86.3% of the total variation range (46.0 days). In contrast to Asia, however, early-flowering accessions from this region are relatively rare. Statistical analyses detected a significant positive correlation between flowering time and latitude in Transcaucasus-Middle East but not between flowering time and winter temperature (Table 3). This suggests that, although early-flowering accessions are rare, some latitudinal environmental factor (e.g., photoperiod length) affected the geographic pattern of flowering time variation in the western part of the species range. Accordingly, mechanisms other than modification of vernalization requirement are assumed to underlie the shortening of flowering time in the southern part of the western habitats.

If photoperiod length were the factor that caused the latitudinal cline, the *Ppd* genes would be the candidates for those responsible for the emergence of early-flowering phenotype in Transcaucasus-Middle East. Because dominant *Ppd* alleles reduce sensitivity to photoperiod length in common wheat [32]–[33], the evolution of western early-flowering *Ae. tauschii* populations might have involved *Ppd* gene mutation that conferred photoperiod insensitivity. The short-flowering-time accessions (i.e., early-flowering and early-intermediate-flowering accessions) of the HG7 lineages seem to have contributed to the postglacial southward dispersal from the TDV zone in this region (Fig. 1). The timing (i.e., before or after the Holocene climate change) of short-flowering-time evolution in this region, however, is difficult to infer from our data.

In conclusion, flowering time diversification has been affected by different environmental factors in the western and eastern parts of the range of *Ae. tauschii*. The postglacial southward dispersal from the TDV zone seems to have been driven by lineages that evolved the short-flowering-time phenotypes. Different genetic mechanisms are probably responsible for the evolution of the short-flowering-time phenotypes in Transcaucasus-Middle East and Asia, whereas those populations that migrated southward had relatively narrow genealogical backgrounds. These findings provide the basis for analyses of genetic mechanisms that underlie flowering time diversification in *Ae. tauschii*. Because admixture may have significant influence on genetic structure even in selfing species [34]–[35], the cpDNA genealogy and inferences about the evolution of the short-flowering-time phenotypes require further testing with nuclear gene sequences. Central Eurasia is the key area for understanding the Asian-European vegetation transition. However, species histories of divergence and dispersal on this landmass are largely unknown [36]. Further studies using *Ae. tauschii* as a model system should help understand how plants adapted to the diverse environments of Eurasia.

## Supporting Information

Table S1 *Ae. tauschii* accessions and their geographic, phenotypic, genetic and palaeoclimatic properties
(0.13 MB PDF)

Table S2 Chloroplast haplogroups and defining biallelic variations
(0.08 MB PDF)

Table S3 Chloroplast haplotypes and defining variations
(0.07 MB PDF)

Figure S1 Phylogenetic relationships between the HG7 and HG16 chloroplast DNA haplotypes (Table S3). A majority-rule consensus tree was obtained from 1,000 bootstrapped neighbor-joining trees. Branch length is proportional to the number of times a branch appeared in the bootstrap samples. The bootstrap values are shown on branches only if they are larger than 500. The cluster of HG16 haplotypes is boxed. The grey branches denote the cluster that includes all eastern habitat haplotypes of HG7.
(0.07 MB PDF)
