# Supplementary material for: Flowering Time Diversification and Dispersal in Central Eurasian Wild Wheat Aegilops tauschii Coss.: Genealogical and Ecological Framework
Source: PLoS One. 2008 Sep 4;3(9):e3138. doi: 10.1371/journal.pone.0003138 (PMC2519791; doi:10.1371/journal.pone.0003138)
Supplement: Table S1 — Ae. tauschii accessions and their geographic, phenotypic, genetic and palaeoclimatic properties (0.13 MB PDF) [file pone.0003138.s001.pdf]

Table S1. *Ae. tauschii* accessions and their geographic, phenotypic, genetic and palaeoclimatic properties

No.	Accession	Latitude	Longitude	Flowering time (day)	Haplogroup[1)]	Haplotype[2)]	Vegetation at the Last Glacial Maximum[3)]	Mean temperature of coldest quarter (˚C)[4)]	Annual precipitation (mm)[4)]
1	AE 454	41.89472	44.80194	172.7	HG17	-	Temperate desert vegetation	0.1500	704.0000
2	AE 457	41.63528	44.89500	180.0	HG17	-	Temperate desert vegetation	2.7000	511.0000
3	AE 929	41.89472	44.80194	173.0	HG17	-	Temperate desert vegetation	0.1500	704.0000
4	AE 933	41.84583	44.79167	163.3	HG7	HT7-7	Temperate desert vegetation	-0.4500	742.0000
5	AE 1037	41.71389	44.35417	166.7	HG7	HT7-11	Temperate desert vegetation	-3.0333	792.0000
6	AE 1038	37.33528	68.29778	144.0	HG7	HT7-15	Temperate desert vegetation	4.2667	293.0000
7	AE 1090	42.09750	69.53361	153.0	HG16	HT16D	Temperate desert vegetation	-0.4167	529.0000
8	CGN 10767	30.41667	66.96667	151.0	HG13	-	Tropiacal semi-desert vegetation	5.8000	255.0000
9	CGN 10768	30.38333	67.00000	150.0	HG16	HT16K	Tropiacal semi-desert vegetation	5.6333	256.0000
10	CGN 10769	30.55250	66.87917	150.7	HG16	HT16J	Tropiacal semi-desert vegetation	6.3833	251.0000
11	CGN 10770	30.24944	67.02944	150.0	HG16	HT16J	Tropiacal semi-desert vegetation	5.2167	248.0000
12	CGN 10771	30.22028	67.01083	151.0	HG16	HT16K	Tropiacal semi-desert vegetation	5.2167	248.0000
13	IG 46623	36.73889	40.27500	167.3	HG9	-	Tropiacal semi-desert vegetation	6.8667	368.0000
14	IG 46663	30.53333	67.25000	156.3	HG16	HT16K	Tropiacal semi-desert vegetation	4.2500	265.0000
15	IG 46666	30.75000	67.55000	154.7	HG16	HT16K	Tropiacal semi-desert vegetation	1.6833	281.0000
16	IG 46682	30.66667	68.66667	151.0	HG16	HT16J	Tropiacal semi-desert vegetation	6.7167	212.0000
17	IG 47173	39.51667	46.36667	190.0	HG7	HT7-35	Temperate desert vegetation	-1.9000	608.0000
18	IG 47182	39.05000	48.66667	162.3	HG7	HT7-35	Temperate desert vegetation	4.2333	656.0000
19	IG 47186	40.08333	49.40000	168.0	HG7	HT7-35	Temperate desert vegetation	4.6500	271.0000
20	IG 47188	40.98333	47.83333	165.3	HG7	HT7-11	Temperate desert vegetation	1.3667	671.0000
21	IG 47192	38.93333	48.25000	166.7	HG7	HT7-35	Temperate desert vegetation	0.3833	424.0000
22	IG 47193	38.75000	48.40000	166.0	HG9	-	Temperate desert vegetation	0.3500	409.0000
23	IG 47194	40.50000	50.00000	174.3	HG7	HT7-35	Temperate desert vegetation	4.8167	249.0000
24	IG 47196	40.38333	49.88333	162.3	HG7	HT7-31	Temperate desert vegetation	5.0167	255.0000
25	IG 47199	40.63333	48.61667	169.7	HG7	HT7-35	Temperate desert vegetation	1.1833	560.0000
26	IG 47202	39.80000	46.75000	167.0	HG7	HT7-35	Temperate desert vegetation	0.8167	548.0000
27	IG 47203	39.38333	47.01667	170.3	HG7	HT7-35	Temperate desert vegetation	2.3333	436.0000
28	IG 47204	41.20000	49.03333	167.0	HG7	HT7-35	Temperate desert vegetation	3.2833	363.0000
29	IG 47259	35.57778	38.82222	150.3	HG7	HT7-18	Tropiacal semi-desert vegetation	7.7667	167.0000
30	IG 48042	34.08333	74.80000	163.0	HG14	-	Tropiacal semi-desert vegetation	3.1333	697.0000
31	IG 48274	42.20000	47.91667	172.0	HG7	HT7-35	Temperate desert vegetation	1.3000	413.0000
32	IG 48508	38.33333	55.86667	157.0	HG7	HT7-15	Temperate desert vegetation	4.5000	226.0000
33	IG 48518	38.45000	56.00000	154.7	HG16	HT16L	Temperate desert vegetation	3.8500	225.0000
34	IG 48539	41.10000	69.00000	153.7	HG16	HT16D	Temperate desert vegetation	3.2167	375.0000
35	IG 48554	39.46667	67.50000	153.3	HG7	HT7-20	Temperate desert vegetation	1.6167	387.0000
36	IG 48559	39.75000	68.63333	161.0	HG16	HT16J	Temperate desert vegetation	-3.4833	687.0000
37	IG 48564	40.08333	69.11667	153.0	HG7	HT7-2	Temperate desert vegetation	1.6833	380.0000
38	IG 48565	40.45000	71.06667	157.7	HG16	HT16D	Temperate desert vegetation	1.1000	249.0000
39	IG 48567	40.56667	71.70000	163.0	HG16	HT16C	Temperate desert vegetation	1.2667	210.0000
40	IG 48747	40.28333	44.63333	175.7	HG7	HT7-15	Temperate desert vegetation	-2.7333	403.0000
41	IG 48748	40.18333	44.66667	162.0	HG10	-	Temperate desert vegetation	-4.4667	438.0000
42	IG 48758	40.25000	44.33333	165.3	HG7	HT7-15	Temperate desert vegetation	-1.7167	337.0000
43	IG 49095	36.33333	47.83333	157.3	HG7	HT7-30	Tropiacal semi-desert vegetation	-1.3000	392.0000
44	IG 108561	29.88333	66.83333	153.3	HG16	HT16J	Tropiacal semi-desert vegetation	6.5833	199.0000
45	IG 120735	38.53333	57.16667	164.7	HG16	HT16H	Temperate desert vegetation	3.6333	205.0000

46	IG 120736	39.91667	66.36667	151.7	HG16	HT16D	Temperate desert vegetation	2.7667	273.0000
47	IG 120863	41.58333	48.28333	169.7	HG7	HT7-34	Temperate desert vegetation	0.9333	476.0000
48	IG 120866	41.88333	48.38333	166.3	HG9	-	Temperate desert vegetation	2.6167	363.0000
49	IG 123910	40.79722	72.36667	159.0	HG16	HT16D	Temperate desert vegetation	0.4333	260.0000
50	IG 126273	39.99722	44.95833	165.0	HG7	HT7-29	Temperate desert vegetation	-3.1500	388.0000
51	IG 126280	39.90278	44.94167	166.3	HG7	HT7-15	Temperate desert vegetation	-4.6667	418.0000
52	IG 126293	39.79722	45.33333	164.3	HG7	HT7-15	Temperate desert vegetation	-2.0833	382.0000
53	IG 126353	39.71111	45.56944	166.3	HG7	HT7-15	Temperate desert vegetation	-2.5500	436.0000
54	IG 126387	38.63889	56.85833	160.7	HG16	HT16O	Temperate desert vegetation	2.8500	210.0000
55	IG 126489	38.01944	58.23611	151.7	HG2	-	Temperate desert vegetation	3.6667	231.0000
56	IG 126991	39.08611	46.58056	171.7	HG9	-	Temperate desert vegetation	1.5000	402.0000
57	IG 127015	39.07778	46.30556	175.3	HG7	HT7-35	Temperate desert vegetation	-6.6500	478.0000
58	IG 131606	42.71667	72.00833	160.3	HG16	HT16L	Temperate desert vegetation	-12.4000	558.0000
59	KU-20-1	42.05778	48.32528	173.3	HG7	HT7-35	Temperate desert vegetation	2.5000	358.0000
60	KU-20-6	30.07889	66.89528	149.7	HG16	HT16J	Tropiacal semi-desert vegetation	2.9000	235.0000
61	KU-20-7	35.84833	51.03722	159.7	HG7	HT7-9	Tropiacal semi-desert vegetation	0.1667	218.0000
62	KU-20-8	35.87444	52.65389	159.7	HG4	-	Tropiacal semi-desert vegetation	-5.4500	159.0000
63	KU-20-9	36.88333	53.46778	163.7	HG3	-	Temperate desert vegetation	8.7333	488.0000
64	KU-20-10	37.03917	50.69306	164.0	HG9	-	Temperate desert vegetation	7.5500	1156.0000
65	KU-2001	30.14528	66.90333	153.7	HG16	HT16E	Tropiacal semi-desert vegetation	3.2167	241.0000
66	KU-2003	30.14528	66.90333	150.0	HG16	HT16J	Tropiacal semi-desert vegetation	3.2167	241.0000
67	KU-2006	30.69000	66.66972	147.7	HG16	HT16J	Tropiacal semi-desert vegetation	6.6833	243.0000
68	KU-2008	31.03444	66.32611	150.0	HG16	HT16J	Tropiacal semi-desert vegetation	8.7333	212.0000
69	KU-2010	31.82778	66.21333	149.7	HG8	-	Tropiacal semi-desert vegetation	4.3500	234.0000
70	KU-2012	32.03083	66.68667	150.0	HG8	-	Tropiacal semi-desert vegetation	1.4167	271.0000
71	KU-2016	32.81250	67.74889	166.3	HG7	HT7-15	Tropiacal semi-desert vegetation	-4.4500	196.0000
72	KU-2018	33.80417	68.40583	157.3	HG16	HT16F	Tropiacal semi-desert vegetation	-5.3667	273.0000
73	KU-2022	34.61861	69.31250	162.0	HG16	HT16L	Tropiacal semi-desert vegetation	0.7667	375.0000
74	KU-2025	35.91333	68.92194	160.0	HG8	-	Temperate desert vegetation	1.1833	474.0000
75	KU-2027	36.15361	68.75083	154.0	HG7	HT7-16	Temperate desert vegetation	4.0833	296.0000
76	KU-2028	36.17611	68.64778	149.3	HG7	HT7-14	Temperate desert vegetation	3.2000	320.0000
77	KU-2032	36.31194	68.59583	148.3	HG7	HT7-15	Temperate desert vegetation	2.9833	326.0000
78	KU-2035	36.21944	68.59472	152.3	HG7	HT7-15	Temperate desert vegetation	3.2000	314.0000
79	KU-2039	36.23833	68.59361	148.0	HG15	-	Temperate desert vegetation	3.2000	314.0000
80	KU-2042	36.20139	68.51639	149.3	HG7	HT7-27	Temperate desert vegetation	1.8667	357.0000
81	KU-2043	37.35444	64.98361	150.3	HG15	-	Temperate desert vegetation	4.3833	240.0000
82	KU-2044	36.08306	65.03167	150.7	HG15	-	Temperate desert vegetation	3.3167	368.0000
83	KU-2050	36.00750	64.78917	148.3	HG16	HT16G	Temperate desert vegetation	3.9667	388.0000
84	KU-2051	35.91333	64.87972	148.0	HG16	HT16G	Temperate desert vegetation	3.1000	357.0000
85	KU-2056	35.94944	64.89500	153.7	HG16	HT16K	Temperate desert vegetation	3.8000	365.0000
86	KU-2058	35.81056	64.59139	151.7	HG7	HT7-16	Temperate desert vegetation	3.2333	366.0000
87	KU-2059	35.73556	64.27333	152.0	HG16	HT16J	Temperate desert vegetation	2.1167	368.0000
88	KU-2061	35.78417	63.94861	147.7	HG16	HT16A	Temperate desert vegetation	3.4500	367.0000
89	KU-2063	35.51111	64.09056	148.7	HG16	HT16L	Temperate desert vegetation	-3.7000	360.0000
90	KU-2066	35.24722	63.46167	147.7	HG16	HT16P	Temperate desert vegetation	4.1667	343.0000
91	KU-2068	36.37750	50.09389	153.0	HG16	HT16K	Tropiacal semi-desert vegetation	0.2833	275.0000
92	KU-2069	35.84833	51.03722	160.7	HG7	HT7-9	Tropiacal semi-desert vegetation	0.1667	218.0000
93	KU-2074	36.88333	53.46778	164.3	HG5	-	Temperate desert vegetation	8.7333	488.0000

94	KU-2075	36.87139	53.73028	167.0	HG12	-	Temperate desert vegetation	8.9500	453.0000
95	KU-2076	37.09500	54.32639	159.3	HG9	-	Temperate desert vegetation	8.7167	389.0000
96	KU-2077	37.19722	54.92944	163.7	HG7	HT7-3	Temperate desert vegetation	8.9667	368.0000
97	KU-2078	37.14000	54.82806	158.3	HG9	-	Temperate desert vegetation	9.0000	377.0000
98	KU-2079	37.14000	54.82806	162.7	HG9	-	Temperate desert vegetation	9.0000	377.0000
99	KU-2080	37.26861	55.10944	160.7	HG9	-	Temperate desert vegetation	8.8167	357.0000
100	KU-2082	37.16583	55.31139	159.7	HG16	HT16N	Temperate desert vegetation	7.5833	341.0000
101	KU-2083	37.09667	55.30194	160.3	HG7	HT7-36	Temperate desert vegetation	6.5833	318.0000
102	KU-2086	35.91611	52.77000	165.5	HG7	HT7-6	Tropiacal semi-desert vegetation	-3.9500	153.0000
103	KU-2087	36.74167	53.28750	159.7	HG16	HT16N	Temperate desert vegetation	8.9500	595.0000
104	KU-2088	36.92250	53.38111	161.3	HG9	-	Temperate desert vegetation	8.5833	498.0000
105	KU-2090	36.85639	53.60750	161.7	HG9	-	Temperate desert vegetation	8.9167	475.0000
106	KU-2091	36.92111	52.60167	158.0	HG18	-	Temperate desert vegetation	8.2000	752.0000
107	KU-2092	36.92111	52.60167	160.7	HG1	-	Temperate desert vegetation	8.2000	752.0000
108	KU-2093	36.86556	52.42139	162.0	HG7	HT7-12	Temperate desert vegetation	8.2500	818.0000
109	KU-2096	36.86556	52.42139	158.7	HG9	-	Temperate desert vegetation	8.2500	818.0000
110	KU-2097	36.86556	52.42139	151.3	HG7	HT7-13	Temperate desert vegetation	8.2500	818.0000
111	KU-2098	36.95444	50.74500	157.3	HG9	-	Temperate desert vegetation	7.6500	1146.0000
112	KU-2100	37.07000	50.47194	162.0	HG9	-	Temperate desert vegetation	7.6833	1165.0000
113	KU-2101	37.16750	50.45694	159.3	HG9	-	Temperate desert vegetation	7.5500	1177.0000
114	KU-2102	37.31972	50.33694	157.7	HG9	-	Temperate desert vegetation	7.4833	1188.0000
115	KU-2103	37.33583	49.72694	157.3	HG9	-	Temperate desert vegetation	7.7500	1358.0000
116	KU-2104	37.58972	49.61556	159.3	HG7	HT7-11	Temperate desert vegetation	7.5167	1426.0000
117	KU-2105	37.66000	49.44472	164.3	HG9	-	Temperate desert vegetation	7.5000	1438.0000
118	KU-2106	37.67417	49.40000	157.0	HG9	-	Temperate desert vegetation	7.4500	1417.0000
119	KU-2107	38.19194	49.00972	166.5	HG9	-	Temperate desert vegetation	6.4500	1245.0000
120	KU-2108	38.19194	49.00972	157.7	HG9	-	Temperate desert vegetation	6.4500	1245.0000
121	KU-2109	38.49056	49.02028	159.3	HG7	HT7-17	Temperate desert vegetation	5.7500	1162.0000
122	KU-2110	38.43472	48.75500	172.0	HG7	HT7-8	Temperate desert vegetation	5.2500	1127.0000
123	KU-2111	38.34667	48.42139	170.0	HG7	HT7-35	Temperate desert vegetation	-0.2167	365.0000
124	KU-2112	38.26111	48.28917	168.0	HG7	HT7-35	Temperate desert vegetation	-0.6500	326.0000
125	KU-2113	36.75806	45.94056	160.3	HG7	HT7-16	Tropiacal semi-desert vegetation	-1.9833	436.0000
126	KU-2115	37.07167	45.74083	158.3	HG7	HT7-15	Tropiacal semi-desert vegetation	0.2667	409.0000
127	KU-2116	38.29417	45.02472	166.0	HG7	HT7-15	Tropiacal semi-desert vegetation	-2.3500	353.0000
128	KU-2118	38.62194	45.10333	160.3	HG4	-	Tropiacal semi-desert vegetation	0.3167	278.0000
129	KU-2120	38.49167	45.87583	159.0	HG7	HT7-17	Tropiacal semi-desert vegetation	-1.4000	338.0000
130	KU-2121	38.37500	46.13444	165.7	HG7	HT7-15	Tropiacal semi-desert vegetation	-2.7667	365.0000
131	KU-2122	38.08417	46.40611	153.7	HG7	HT7-33	Tropiacal semi-desert vegetation	-1.1667	334.0000
132	KU-2124	36.71722	51.46000	153.0	HG4	-	Temperate desert vegetation	7.9667	1092.0000
133	KU-2126	36.71722	51.46000	154.0	HG4	-	Temperate desert vegetation	7.9667	1092.0000
134	KU-2131	38.29222	43.15306	167.3	HG7	HT7-14	Tropiacal semi-desert vegetation	-1.5333	518.0000
135	KU-2132	38.29222	43.15306	166.3	HG7	HT7-14	Tropiacal semi-desert vegetation	-1.5333	518.0000
136	KU-2133	38.63556	43.82889	163.0	HG7	HT7-14	Tropiacal semi-desert vegetation	-5.7167	449.0000
137	KU-2136	38.91944	43.62222	164.7	HG7	HT7-14	Tropiacal semi-desert vegetation	-3.3000	460.0000
138	KU-2137	39.06583	43.53556	165.7	HG7	HT7-14	Tropiacal semi-desert vegetation	-4.5167	473.0000
139	KU-2138	39.03917	43.51389	167.0	HG7	HT7-14	Tropiacal semi-desert vegetation	-4.1167	469.0000
140	KU-2140	39.03917	43.51389	165.7	HG7	HT7-14	Tropiacal semi-desert vegetation	-4.1167	469.0000
141	KU-2141	38.94278	43.40583	164.7	HG7	HT7-14	Tropiacal semi-desert vegetation	-3.0500	456.0000

142	KU-2142	39.31667	44.84472	169.0	HG7	HT7-14	Tropiacal semi-desert vegetation	-0.2000	257.0000
143	KU-2143	39.25944	45.06583	164.3	HG7	HT7-14	Tropiacal semi-desert vegetation	0.2000	258.0000
144	KU-2144	39.24278	45.16333	167.7	HG10	-	Tropiacal semi-desert vegetation	0.3000	257.0000
145	KU-2145	39.10028	45.24222	161.7	HG7	HT7-16	Tropiacal semi-desert vegetation	-0.0500	263.0000
146	KU-2148	38.74583	45.89806	162.7	HG7	HT7-15	Tropiacal semi-desert vegetation	-5.5500	420.0000
147	KU-2149	38.90278	45.75583	162.0	HG7	HT7-15	Tropiacal semi-desert vegetation	1.3667	283.0000
148	KU-2150	38.79639	45.77444	162.0	HG7	HT7-15	Tropiacal semi-desert vegetation	-1.4000	349.0000
149	KU-2151	37.43806	47.66778	158.3	HG7	HT7-19	Tropiacal semi-desert vegetation	2.0500	419.0000
150	KU-2152	37.15028	47.92639	161.0	HG7	HT7-28	Tropiacal semi-desert vegetation	2.1500	430.0000
151	KU-2153	36.32806	50.14722	159.0	HG7	HT7-6	Tropiacal semi-desert vegetation	2.0833	293.0000
152	KU-2154	35.98694	49.60972	156.0	HG16	HT16K	Tropiacal semi-desert vegetation	3.3333	307.0000
153	KU-2155	35.63250	49.39611	159.0	HG4	-	Tropiacal semi-desert vegetation	-0.1500	285.0000
154	KU-2156	35.63250	49.39611	159.7	HG4	-	Tropiacal semi-desert vegetation	-0.1500	285.0000
155	KU-2157	34.15750	46.56167	162.0	HG7	HT7-32	Tropiacal semi-desert vegetation	2.6833	479.0000
156	KU-2158	36.95444	50.74500	160.0	HG7	HT7-5	Temperate desert vegetation	7.6500	1146.0000
157	KU-2159	36.95444	50.74500	158.0	HG7	HT7-5	Temperate desert vegetation	7.6500	1146.0000
158	KU-2160	36.95444	50.74500	165.7	HG9	-	Temperate desert vegetation	7.6500	1146.0000
159	KU-2612	34.64000	68.95833	164.3	HG16	HT16F	Tropiacal semi-desert vegetation	-8.3667	736.0000
160	KU-2616	36.61250	70.12444	154.0	HG16	HT16D	Temperate desert vegetation	-9.1833	983.0000
161	KU-2617	36.63972	70.07222	154.7	HG7	HT7-1	Temperate desert vegetation	-4.2833	821.0000
162	KU-2619	37.19472	71.26667	166.3	HG16	HT16B	Temperate desert vegetation	-10.8167	652.0000
163	KU-2621	37.19194	71.24000	167.7	HG16	HT16B	Temperate desert vegetation	-13.0667	756.0000
164	KU-2624	36.87889	71.39167	166.0	HG16	HT16J	Temperate desert vegetation	-16.5000	832.0000
165	KU-2627	36.79611	71.16194	166.3	HG16	HT16I	Temperate desert vegetation	-10.0333	713.0000
166	KU-2630	37.01556	71.59806	168.0	HG11	-	Temperate desert vegetation	-15.3167	730.0000
167	KU-2632	36.97806	71.59972	170.0	HG16	HT16L	Temperate desert vegetation	-15.9833	753.0000
168	KU-2633	36.93361	71.44556	164.7	HG7	HT7-14	Temperate desert vegetation	-11.5333	656.0000
169	KU-2635	36.88222	71.54778	165.0	HG16	HT16J	Temperate desert vegetation	-10.3500	583.0000
170	KU-2636	36.61972	71.49667	165.3	HG16	HT16D	Temperate desert vegetation	-12.0833	708.0000
171	KU-2638	36.57083	71.53611	167.3	HG16	HT16F	Temperate desert vegetation	-15.8667	799.0000
172	KU-2639	36.40028	69.11222	148.0	HG16	HT16M	Temperate desert vegetation	2.0333	462.0000
173	KU-2801	40.65639	49.78861	167.0	HG7	HT7-35	Temperate desert vegetation	4.8000	297.0000
174	KU-2804	40.58500	48.85250	168.0	HG7	HT7-9	Temperate desert vegetation	0.7500	506.0000
175	KU-2806	40.58500	48.85250	166.7	HG9	-	Temperate desert vegetation	0.7500	506.0000
176	KU-2809	40.24528	44.62111	172.7	HG7	HT7-16	Temperate desert vegetation	-2.6167	385.0000
177	KU-2810	40.24528	44.62111	162.3	HG10	-	Temperate desert vegetation	-2.6167	385.0000
178	KU-2811	40.24528	44.62111	159.3	HG4	-	Temperate desert vegetation	-2.6167	385.0000
179	KU-2814	40.24528	44.62111	162.7	HG10	-	Temperate desert vegetation	-2.6167	385.0000
180	KU-2816	40.22028	44.55194	162.7	HG10	-	Temperate desert vegetation	-2.2667	365.0000
181	KU-2821	40.19528	44.74778	165.7	HG7	HT7-25	Temperate desert vegetation	-5.0000	454.0000
182	KU-2822A	40.39139	44.26861	160.7	HG7	HT7-4	Temperate desert vegetation	-6.6167	508.0000
183	KU-2823	40.38333	44.29806	161.3	HG7	HT7-24	Temperate desert vegetation	-5.6333	480.0000
184	KU-2824	40.38333	44.29806	160.7	HG10	-	Temperate desert vegetation	-5.6333	480.0000
185	KU-2826	41.54806	45.10139	174.7	HG7	HT7-23	Temperate desert vegetation	2.9833	507.0000
186	KU-2827	41.75944	44.85167	168.0	HG9	-	Temperate desert vegetation	1.6667	585.0000
187	KU-2828	41.84000	44.93861	168.0	HG7	HT7-22	Temperate desert vegetation	-1.6167	840.0000
188	KU-2829A	41.82333	44.81750	172.7	HG17	-	Temperate desert vegetation	1.9500	569.0000
189	KU-2832	41.82333	44.81750	171.0	HG17	-	Temperate desert vegetation	1.9500	569.0000

190	KU-2834	41.82333	44.81750	164.7	HG7	HT7-10	Temperate desert vegetation	1.9500	569.0000
191	KU-2835B	42.08694	44.48472	174.3	HG9	-	Temperate desert vegetation	-0.9000	804.0000
192	KU-2836	42.06556	44.26278	172.0	HG7	HT7-21	Temperate desert vegetation	-0.3833	693.0000
193	PI 476874	36.08306	65.03167	149.3	HG15	-	Temperate desert vegetation	3.3167	368.0000
194	PI 486267	37.20000	44.61667	162.3	HG4	-	Tropiacal semi-desert vegetation	-6.5000	595.0000
195	PI 486270	37.78333	44.33333	162.7	HG7	HT7-15	Tropiacal semi-desert vegetation	-9.6000	547.0000
196	PI 486274	40.15000	43.36667	169.3	HG7	HT7-14	Tropiacal semi-desert vegetation	-5.1667	404.0000
197	PI 486277	40.08333	42.93333	172.0	HG7	HT7-5	Tropiacal semi-desert vegetation	-7.4000	510.0000
198	PI 499262	44.00000	81.00000	171.7	HG6	-	Temperate desert vegetation	-7.1333	261.0000
199	PI 508262	44.00000	81.00000	180.7	HG6	-	Temperate desert vegetation	-7.1333	261.0000
200	PI 554319	37.48333	43.71667	163.7	HG7	HT7-26	Tropiacal semi-desert vegetation	-3.2833	746.0000

Longitude and latitude are given as decimal degrees. See text for details of cpDNA haplotypes. AE accessions were provided by Institut für Pflanzengenetik und Kulturpflanzenforschung; AT accessions by Okayama University; CGN accessions by Centre for Genetic Resources, The Netherlands; IG accessions by International Center for Agricultural Research in the Dry Areas; KU accessions by Plant Germ-plasm Institute, Kyoto University; and PI accessions by United States Department of Agriculture.

1) Chloroplast DNA haplogroups based on variations at 21 biallelic sites (Table S2).

2) Chloroplast DNA haplotypes based on variations at 14 loci (Table S3).

3) Vegetations 25,000-15,000BP for the sampling sites (estimated from [28]).

4) Estimated using the DIVA-GIS software [21].
