# Supplementary material for: Flowering Time Diversification and Dispersal in Central Eurasian Wild Wheat Aegilops tauschii Coss.: Genealogical and Ecological Framework
Source: PLoS One. 2008 Sep 4;3(9):e3138. doi: 10.1371/journal.pone.0003138 (PMC2519791; doi:10.1371/journal.pone.0003138)
Supplement: Table S2 — Chloroplast haplogroups and defining biallelic variations (0.08 MB PDF) [file pone.0003138.s002.pdf]

**Table S2.** Chloroplast haplogroups and defining biallelic variations

| cpDNA Haplogroup | No. of accessions | Haplogroup-defining-site name | | | | | | | | | | | | | | | | | | | | |
|---|---|---|---|---|---|---|---|---|---|---|---|---|---|---|---|---|---|---|---|---|---|---|
| | | WCt6c (109) | WCt6d (113) | WCt6e (119) | WCt10b (141) | WCt10c (97) | WCt10d (83) | WCt10e (32) | WCt10f (30) | WCt17b (56) | WCt17c (345) | WCt24b (7) | WC24c (33) | WCt24e (37) | WCt24f (40) | WCt24g (41) | WCt24h (31) | WCt24i (99) | WCt24j (112) | *trn*LFb (154) | *trn*TLa (202) | *trn*TLb (192) |
| HG1 | 1 | G | G | A | C | A | A | A | T | TTC | G | TAAAAAA | T | T | C | T | 2 | T | G | T | 1 | G |
| HG2 | 1 | G | G | A | C | A | A | T | G | TTC | G | TAAAAAA | T | T | C | T | 1 | T | G | C | 1 | G |
| HG3 | 1 | G | G | A | C | A | A | T | T | TTC | A | TAAAAAA | T | T | C | T | 2 | T | G | T | 1 | G |
| HG4 | 8 | G | G | A | C | A | A | T | T | TTC | G | TAAAAAA | A | A | G | A | 1 | T | G | T | 1 | G |
| HG5 | 1 | G | G | A | C | A | A | T | T | TTC | G | TAAAAAA | T | T | C | T | 1 | T | A | T | 1 | G |
| HG6 | 2 | G | G | A | C | A | A | T | T | TTC | G | TAAAAAA | T | T | C | T | 1 | T | G | C | 1 | G |
| HG7 | 90 | G | G | A | C | A | A | T | T | TTC | G | TAAAAAA | T | T | C | T | 1 | T | G | T | 1 | G |
| HG8 | 3 | G | G | A | C | A | A | T | T | TTC | G | TAAAAAA | T | T | C | T | 1 | T | G | T | 2 | C |
| HG9 | 25 | G | G | A | C | A | A | T | T | TTC | G | TAAAAAA | T | T | C | T | 2 | T | G | T | 1 | G |
| HG10 | 6 | G | G | A | C | A | A | T | T | TTC | G | ATCTTTATTTA | T | T | C | T | 1 | T | G | T | 1 | G |
| HG11 | 1 | G | G | A | C | A | C | T | T | TTC | G | TAAAAAA | T | T | C | T | 1 | T | G | T | 1 | G |
| HG12 | 1 | G | G | A | C | C | A | T | T | TTC | G | TAAAAAA | T | T | C | T | 1 | T | G | T | 1 | G |
| HG13 | 1 | G | G | A | T | A | A | T | T | TTC | G | TAAAAAA | T | T | C | T | 1 | T | G | T | 2 | C |
| HG14 | 1 | G | G | C | C | A | A | T | T | TTC | G | TAAAAAA | T | T | C | T | 1 | T | G | C | 1 | G |
| HG15 | 4 | G | T | A | C | A | A | T | T | AAA | G | TAAAAAA | T | T | C | T | 1 | T | G | T | 1 | G |
| HG16 | 48 | G | T | A | C | A | A | T | T | TTC | G | TAAAAAA | T | T | C | T | 1 | T | G | T | 1 | G |
| HG17 | 5 | T | G | A | C | A | A | T | T | TTC | G | TAAAAAA | T | T | C | T | 1 | G | G | T | 1 | G |
| HG18 | 1 | T | G | A | C | A | A | T | T | TTC | G | TAAAAAA | T | T | C | T | 2 | T | G | T | 1 | G |

Site positions, based on aligned sequences, counted from the nucleotide next to the 3’ end of the forward primer are in parentheses.

The WCt17 locus was amplified using WCt17 forward and WCt18 reverse primers [14].

WCt24h has one or two repeats of the unit minisatellite CTTCGTTACCTAGTTATTTT.

*trn*LFb is in the intergenic spacer region between the *trn*L (UAA) and *trn*F (GAA) genes [16].

*trn*TLa and *trn*TLb are in the intergenic spacer region between the *trn*T (UGU) and *trn*L (UAA) genes [16].

*trn*TLa has one or two repeats of the unit sequence ATTTTT.
