# Supplementary material for: Flowering Time Diversification and Dispersal in Central Eurasian Wild Wheat Aegilops tauschii Coss.: Genealogical and Ecological Framework
Source: PLoS One. 2008 Sep 4;3(9):e3138. doi: 10.1371/journal.pone.0003138 (PMC2519791; doi:10.1371/journal.pone.0003138)
Supplement: Table S3 — Chloroplast haplotypes and defining variations (0.07 MB PDF) [file pone.0003138.s003.pdf]

Table S3. Chloroplast haplotypes and defining variations

Haplotype	No. of accessions	Locus name													
		WCt1 (19, A)	WCt4 (39, T)	WCt5 (19, A)	WCt6a (31, T)	WCt6b (78, C)	WCt6d (113)	WCt8 (44, T)	WCt10a (49, A)	WCt12 (38, T)	WCt17a (39, T)	WCt19 (34, A)	WCt24a (104, A)	WCt24d (38)	*trn*LFc (276)
HT7-1	1	9	9	12	9	9	G	8	9	12	9	8	9	C	TTTAAAA
HT7-2	1	9	9	12	9	9	G	8	9	12	10	8	9	C	TTTAAAA
HT7-3	1	9	9	15	9	8	G	8	10	11	12	8	9	C	TTTTAAA
HT7-4	1	10	9	10	9	8	G	8	10	10	10	8	10	C	TTTAAAA
HT7-5	3	10	9	10	9	8	G	8	10	10	10	8	11	C	TTTAAAA
HT7-6	2	10	9	10	9	8	G	8	10	10	10	8	AAAAATAAAAA	C	TTTTAAA
HT7-7	1	10	9	10	9	8	G	8	10	10	10	9	11	C	TTTAAAA
HT7-8	1	10	9	10	9	9	G	8	10	10	9	8	8	T	TTTTAAA
HT7-9	3	10	9	11	8	8	G	8	10	11	11	8	9	C	TTTTAAA
HT7-10	1	10	9	11	9	8	G	8	10	10	10	8	11	C	TTTAAAA
HT7-11	3	10	9	11	9	8	G	8	10	11	10	8	9	T	TTTAAAA
HT7-12	1	10	9	11	9	10	G	8	10	10	12	8	9	T	TTTAAAA
HT7-13	1	10	9	11	10	8	G	8	10	11	12	8	9	T	TTTAAAA
HT7-14	13	10	9	12	9	9	G	8	9	12	10	8	9	C	TTTAAAA
HT7-15	17	10	9	12	9	9	G	8	9	12	11	8	9	C	TTTAAAA
HT7-16	5	10	9	12	9	9	G	8	9	12	12	8	9	C	TTTAAAA
HT7-17	2	10	9	12	9	9	G	8	9	12	12	8	9	C	TTTTAAA
HT7-18	1	10	9	12	9	9	G	8	9	12	13	8	9	C	TTTAAAA
HT7-19	1	10	9	12	9	8	G	8	10	10	10	8	11	C	TTTAAAA
HT7-20	1	10	9	12	9	9	G	8	10	12	10	8	9	C	TTTAAAA
HT7-21	1	10	9	13	9	8	G	9	10	10	11	8	9	C	TTTTAAA
HT7-22	1	10	9	13	9	8	G	9	10	9	12	8	9	C	TTTTAAA
HT7-23	1	10	9	13	9	8	G	9	10	10	11	8	9	C	TTTTAAA
HT7-24	1	10	9	13	9	9	G	8	9	12	12	8	9	C	TTTAAAA
HT7-25	1	10	9	13	9	9	G	8	10	11	11	8	9	C	TTTTAAA
HT7-26	1	10	9	13	9	9	G	8	10	11	12	8	9	C	TTTTAAA
HT7-27	1	10	9	13	10	9	G	8	9	12	11	8	9	C	TTTAAAA
HT7-28	1	10	9	14	9	8	G	8	10	11	12	8	10	C	TTTTAAA
HT7-29	1	10	9	14	9	8	G	8	10	11	13	8	9	C	TTTTAAA
HT7-30	1	10	9	14	9	8	G	8	10	12	12	8	10	C	TTTTAAA
HT7-31	1	10	9	14	9	8	G	9	10	10	11	8	9	C	TTTTAAA
HT7-32	1	10	9	15	9	9	G	8	10	11	12	8	9	C	TTTTAAA
HT7-33	1	10	9	16	9	8	G	8	10	11	10	8	10	C	TTTTAAA
HT7-34	1	10	10	11	8	8	G	8	11	11	9	8	10	C	TTTTAAA
HT7-35	15	10	10	11	8	8	G	8	11	11	9	8	11	C	TTTTAAA
HT7-36	1	10	10	12	8	8	G	8	11	11	9	8	11	C	TTTTAAA
HT16A	1	10	9	10	9	8	T	8	10	10	11	8	11	C	TTTTAAA
HT16B	2	10	9	10	9	8	T	8	10	10	11	8	10	C	TTTTAAA
HT16C	1	10	9	11	8	8	T	8	10	11	10	8	9	C	TTTAAAA
HT16D	7	10	9	11	9	8	T	8	9	10	10	8	9	C	TTTTAAA
HT16E	1	10	9	11	9	8	T	8	10	10	9	8	9	C	TTTAAAA
HT16F	3	10	9	11	9	8	T	8	10	10	10	8	10	C	TTTTAAA
HT16G	2	11	9	11	9	8	T	8	10	10	10	8	10	C	TTTTAAA
HT16H	1	10	9	11	9	8	T	8	10	10	10	8	11	C	TTTTAAA
HT16I	1	10	9	11	9	8	T	8	10	10	11	8	10	C	TTTTAAA
HT16J	12	10	9	11	9	8	T	8	10	11	9	8	9	C	TTTAAAA
HT16K	7	10	9	11	9	8	T	8	10	11	9	8	10	C	TTTAAAA

HT16L	5	10	9	11	9	8	T	8	10	11	10	8	9	C	TTTAAAA
HT16M	1	10	9	12	9	8	T	8	9	10	11	8	10	C	TTTTAAA
HT16N	2	10	9	12	9	8	T	8	10	10	9	8	11	C	TTTTAAA
HT16O	1	10	9	12	9	8	T	8	10	11	11	9	9	C	TTTAAAA
HT16P	1	10	9	13	9	8	T	8	10	11	10	8	9	C	TTTTAAA

Locus positions, based on aligned sequences, counted from the nucleotide next to the 3’ end of the forward primer are in parentheses. For microsatellite loci, their repeat units also are given.

The WCt17 locus was amplified using WCt17 forward and WCt18 reverse primers [14].

*trn*LFc is in the intergenic spacer region between the *trn*L (UAA) and *trn*F (GAA) genes [16].
