# Supplementary figures and images for: Flowering Time Diversification and Dispersal in Central Eurasian Wild Wheat Aegilops tauschii Coss.: Genealogical and Ecological Framework
Source: PLoS One. 2008 Sep 4;3(9):e3138. doi: 10.1371/journal.pone.0003138 (PMC2519791; doi:10.1371/journal.pone.0003138)

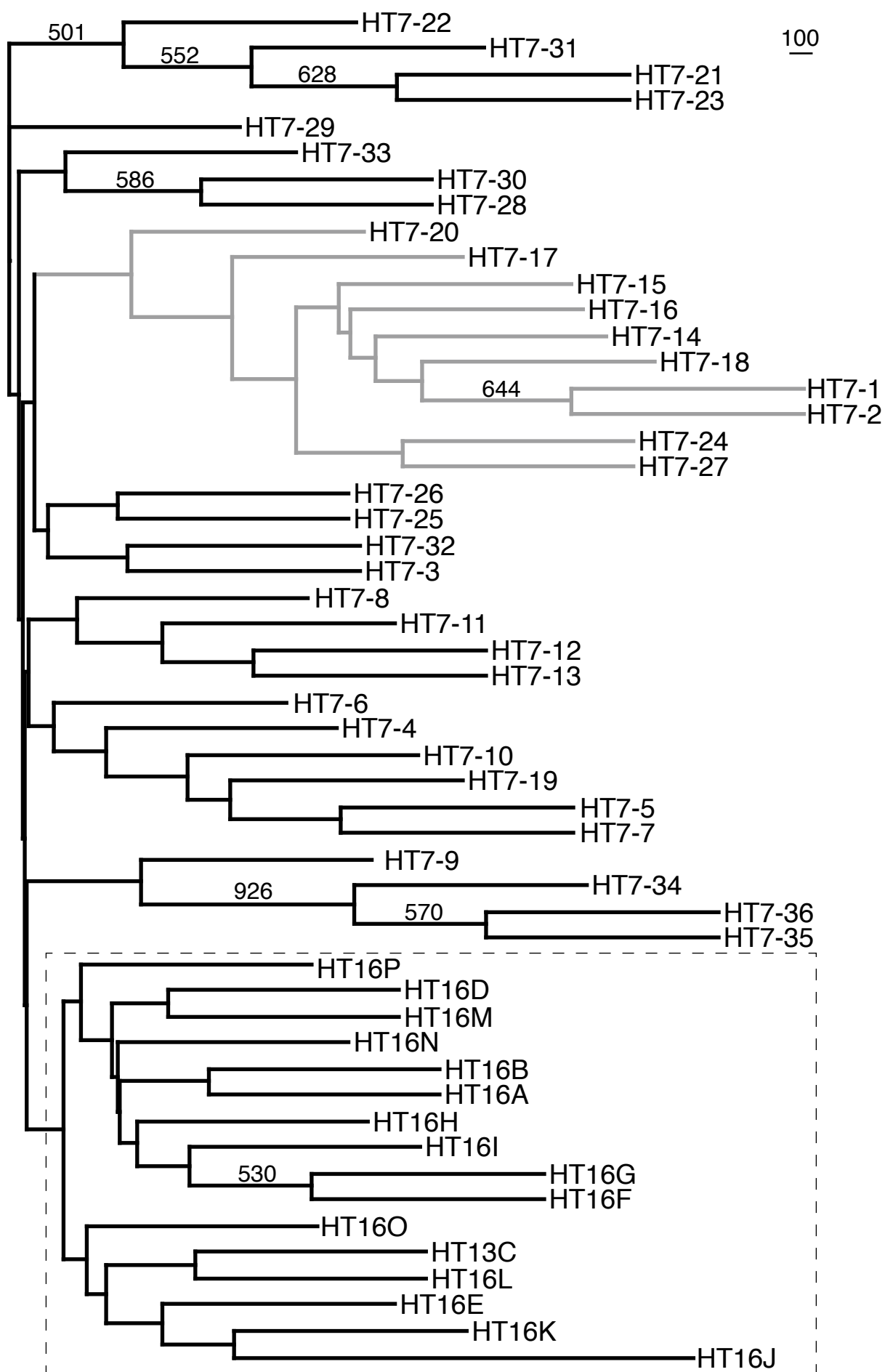

Supplement: Figure S1 — Phylogenetic relationships between the HG7 and HG16 chloroplast DNA haplotypes (Table S3). A majority-rule consensus tree was obtained from 1,000 bootstrapped neighbor-joining trees. Branch length is proportional to the number of times a branch appeared in the bootstrap samples. The bootstrap values are shown on branches only if they are larger than 500. The cluster of HG16 haplotypes is boxed. The grey branches denote the cluster that includes all eastern habitat haplotypes of HG7. (0.07 MB PDF) [file pone.0003138.s004.pdf]
